# Bilateral Renal Infarction as the Initial Manifestation of Left Atrial Myxoma During Early Pregnancy: A Case Report

**DOI:** 10.7759/cureus.117088

**Published:** 2026-09-28

**Authors:** Seung-Do Min, Stanislavs Savjonoks

**Affiliations:** 1 General Internal Medicine, HOCH Health Ostschweiz, Spital Linth, Uznach, CHE; 2 Cardiology, HOCH Health Ostschweiz, Spital Linth, Uznach, CHE

**Keywords:** cardiac myxoma, cardiac surgery, pregnancy, renal infarction, systemic embolism, transthoracic echocardiography

## Abstract

Cardiac myxoma during pregnancy is exceptionally rare and may present with systemic embolisation. A 25-year-old woman at six weeks and five days of gestation presented with persistent central abdominal pain radiating to both flanks. Laboratory investigations showed elevated C-reactive protein (CRP) and lactate dehydrogenase (LDH) levels, while renal function and urinalysis were unremarkable. Non-contrast abdominal magnetic resonance imaging (MRI) demonstrated bilateral renal infarctions. Transthoracic echocardiography subsequently identified a large, mobile, pedunculated left atrial mass, and cardiovascular magnetic resonance (CMR) showed an atypical attachment to the Coumadin ridge. Following multidisciplinary counselling regarding maternal and foetal risks, the patient chose termination of pregnancy followed by surgical resection. Histopathological examination confirmed a left atrial myxoma. The postoperative course was favourable, with preserved renal function and no tumour recurrence on 10-month echocardiographic follow-up. This case highlights bilateral renal infarction as an uncommon initial manifestation of cardiac myxoma and the importance of considering a cardiac embolic source in unexplained visceral infarction during pregnancy.

## Introduction

Primary cardiac tumours are rare, with cardiac myxoma being the most frequently reported benign primary cardiac tumour in adults [1,2]. Most cardiac myxomas arise in the left atrium and are typically attached by a stalk to the region of the fossa ovalis of the interatrial septum [3]. The clinical presentation of cardiac myxoma is highly variable and is commonly classified into obstructive, embolic and constitutional manifestations [1,4]. Systemic embolisation most frequently affects the cerebral circulation, although coronary, retinal, peripheral and visceral embolic events have also been reported [4].

The diagnosis of cardiac myxoma during pregnancy is exceptionally rare [5,6]. Only a few case reports and case series have been published to date, and the 2025 European Society of Cardiology Guidelines for the management of cardiovascular disease during pregnancy do not specifically address cardiac myxoma [7]. The haemodynamic and prothrombotic changes during pregnancy may contribute to the clinical manifestation of a previously silent myxoma, although the precise mechanisms remain uncertain [5,7].

Abdominal and flank pain are prevalent symptoms during pregnancy, with a broad differential diagnosis including obstetric, gastrointestinal and urinary tract causes [8]. Therefore, visceral infarctions may not initially be suspected [8,9]. Acute renal infarction itself is uncommon and often presents non-specifically with abdominal or flank pain, elevated lactate dehydrogenase (LDH), inflammatory markers and haematuria of variable frequency [9].

Management during pregnancy is particularly challenging because the risks of tumour-related cardiac cavity obstruction and systemic embolisation must be balanced against the maternal and foetal risks associated with cardiac surgery and cardiopulmonary bypass [5,7,10]. We report an unusual case of left atrial myxoma arising from the Coumadin ridge during early pregnancy, in which abdominal and flank pain reflected bilateral renal infarction as the first manifestation of systemic embolisation. This case highlights an uncommon embolic presentation and the complexity of clinical decision-making during early pregnancy.

## Case presentation

A 25-year-old woman (gravida II, para I) presented to the emergency department at six weeks and five days gestation with a two- to three-day history of persistent central abdominal pain radiating to both flanks. Her medical history was otherwise unremarkable, and her previous pregnancy had resulted in an uncomplicated spontaneous vaginal delivery. She had no history of autoimmune disease or malignancy and was taking no regular medication other than vitamin supplements. There was no family history of autoimmune or thromboembolic diseases.

The chronological sequence of diagnostic and therapeutic events throughout the patient's clinical course is illustrated in Figure 1.

**Figure 1:**
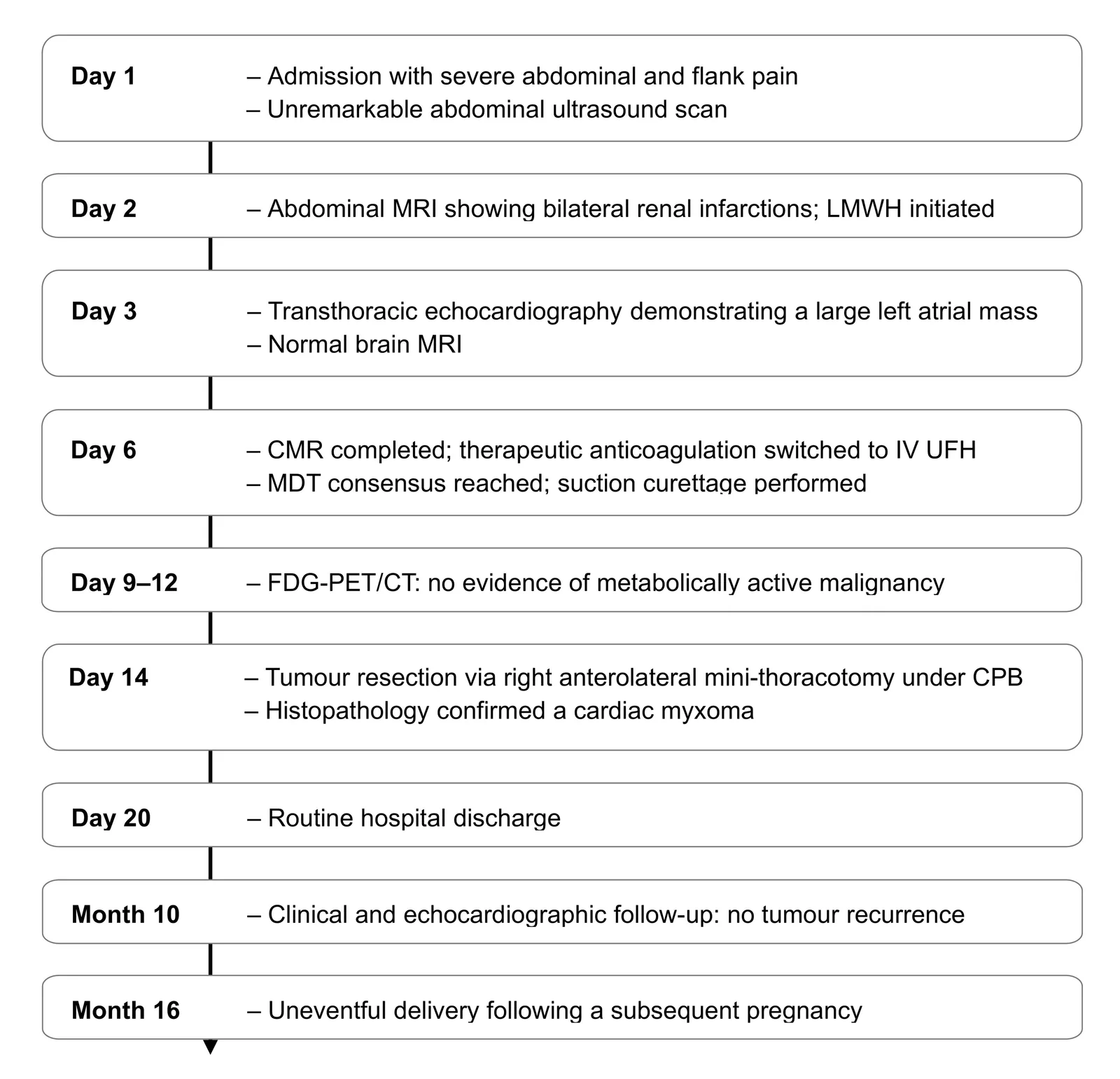
Clinical timeline CMR: cardiovascular magnetic resonance, CPB: cardiopulmonary bypass, IV UFH: intravenous unfractionated heparin, LMWH: low-molecular-weight heparin, MDT: multidisciplinary team, FDG-PET/CT: fluorodeoxyglucose positron emission tomography/computed tomography

The clinical examination revealed no significant findings. The pain responded well to paracetamol and intravenous morphine. Initial obstetric assessment revealed no evidence of pregnancy-related complications, and an internal medicine consultation was requested for further evaluation. On admission, the patient was haemodynamically stable and afebrile. Laboratory investigations revealed a markedly elevated C-reactive protein (CRP) level of 151 mg/L (reference range: <5 mg/L) and a white blood cell count of 17.1 × 10⁹/L (reference range: 4-10 × 10⁹/L). Renal function was preserved with a serum creatinine level of 45 µmol/L (reference range: <95 μmol/L) and an estimated glomerular filtration rate (eGFR) of 133 mL/min/1.73 m² (reference range: >60 mL/min/1.73 m²). Urinalysis was unremarkable, with no proteinuria, bacteriuria or haematuria. The next day, the serum LDH level was elevated to 543 U/L (reference range: 125-220 U/L). No genetic testing for a hereditary predisposition to cardiac myxoma was performed during the reported evaluation.

No significant findings were observed on abdominal ultrasound scan on the day of admission. To avoid foetal exposure to ionising radiation, non-contrast abdominal magnetic resonance imaging (MRI) was performed. This identified bilateral wedge-shaped, inhomogeneous renal lesions consistent with renal infarctions (Figure 2A).

**Figure 2:**
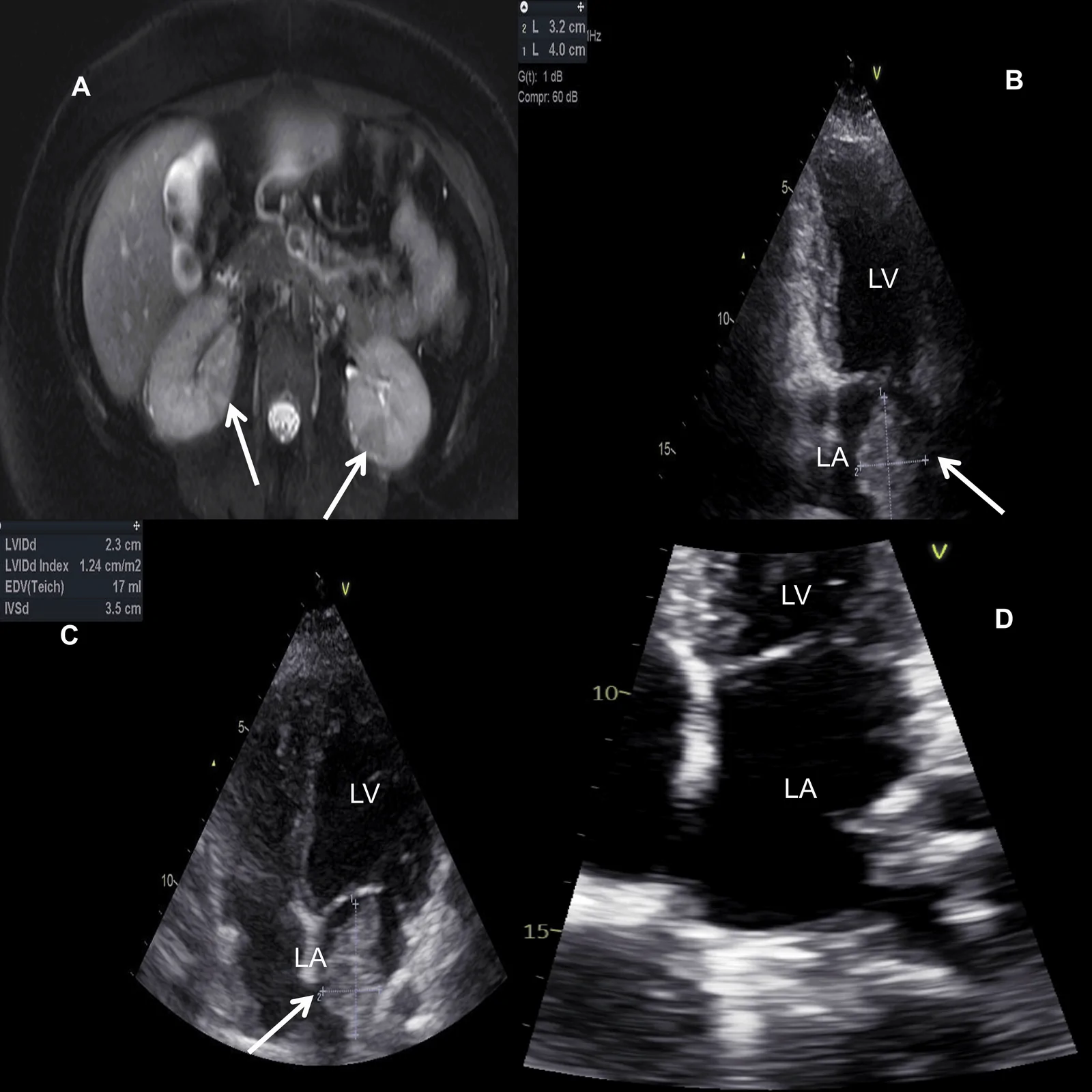
Multimodality imaging findings (A) Preoperative abdominal MRI scan on the day after admission (arrows highlight bilateral renal infarctions). (B and C) Preoperative transthoracic echocardiography with the LV and LA. The arrow indicates the myxoma within the left atrium, measuring approximately 3.2 × 4.0 cm in the depicted imaging plane. (D) Postoperative 10-month follow-up transthoracic echocardiography demonstrating the LV and LA after complete tumour resection. LA: left atrium, LV: left ventricle, MRI: magnetic resonance imaging

In view of these findings, early pregnancy and the need for specialised management, the patient was transferred to a tertiary referral centre for further evaluation of a possible thromboembolic or systemic inflammatory process. Potential cardiac sources of systemic embolisation, including intracardiac thrombus and cardiac tumours, as well as thrombophilia and systemic inflammatory diseases, were considered. The absence of clinical features suggestive of autoimmune disease, negative serological testing and unremarkable thrombophilia screening made inflammatory and prothrombotic causes less likely.

Transthoracic echocardiography showed preserved left ventricular systolic function and a large, mobile, pedunculated mass measuring 2 × 3 × 4 cm within the left atrium. The tumour prolapsed towards the mitral valve during diastole and was considered most consistent with a left atrial myxoma, although an organised thrombus remained in the differential diagnosis (Figure 2B and Figure 2C).

Non-contrast cranial MRI performed on the same day demonstrated no evidence of intracranial haemorrhage or cerebral ischaemia. Further evaluation with cardiovascular magnetic resonance (CMR) demonstrated a solid, lobulated, pedunculated left atrial mass originating from the Coumadin ridge (Figure 3A and Figure 3B).

**Figure 3:**
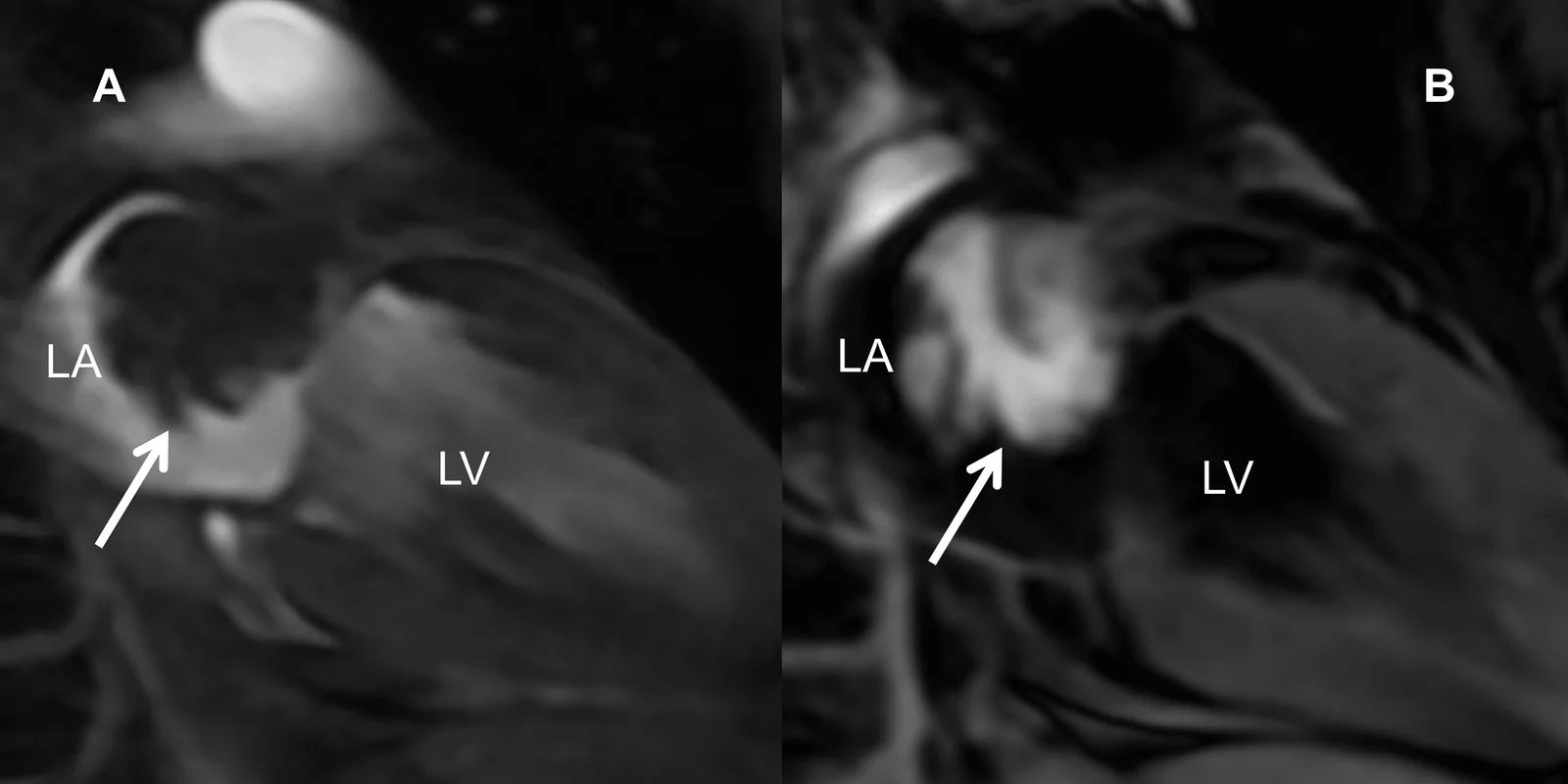
Cardiovascular magnetic resonance findings of the left atrial mass (A) Preoperative cine fast gradient-recalled echo image in the two-chamber view demonstrating a large, lobulated, pedunculated mass within the left atrium (arrow). (B) T2-weighted short tau inversion recovery image in the corresponding two-chamber view demonstrating heterogeneous T2 signal within the mass (arrow), compatible with a myxoma. LA: left atrium, LV: left ventricle

Although tissue characteristics on T2-weighted imaging were compatible with a myxoma, the anatomical location was interpreted as atypical. Consequently, differential diagnoses also included other primary cardiac tumours, such as papillary fibroelastoma and lymphoma.

Following multidisciplinary discussion involving cardiology, cardiac surgery, obstetrics and angiology, the available management options, including termination of pregnancy before cardiac surgery and continuation of the pregnancy with surgical removal of the myxoma, were discussed with the patient. After counselling regarding the maternal and foetal risks, the patient chose termination of pregnancy followed by definitive tumour resection. Therapeutic anticoagulation with low-molecular-weight heparin was initiated because of the presumed bilateral renal infarctions in the setting of a left atrial mass. The treatment was then switched to continuous intravenous unfractionated heparin for closer peri-procedural control before uterine evacuation and cardiac surgery. After informed consent was obtained, suction curettage was performed without complications.

The patient was subsequently transferred to the tertiary cardiac surgery centre for definitive operative management. As part of the preoperative work-up, fluorodeoxyglucose positron emission tomography/computed tomography (FDG-PET/CT) was conducted to assess metabolically active malignancy. Surgical resection of the tumour was performed two weeks after admission via right anterolateral mini-thoracotomy under cardiopulmonary bypass. Histopathological examination of the resected specimen confirmed a left atrial myxoma with haemorrhagic and regressive changes, showing no evidence of malignancy.

The postoperative course was uneventful except for a catheter-associated dissection with partial thrombosis of the left radial artery, which occurred as a procedure-related complication and was successfully managed conservatively. Renal function remained normal throughout follow-up. Transthoracic echocardiography 10 months after surgery showed preserved left ventricular systolic function (left ventricular ejection fraction: 54%) and no evidence of residual or recurrent intra-atrial tumour or clinically relevant structural heart disease (Figure 2D). The patient subsequently conceived again and underwent a successful spontaneous vaginal delivery 16 months after the initial surgical intervention.

## Discussion

We report a rare case of left atrial myxoma in pregnancy, distinguished by three key features. First, the tumour became clinically apparent during the first trimester. A systematic review by Yuan found that only 7 of 37 (18.9%) patients were diagnosed during the first trimester, compared with 19 (51.4%) in the second and 11 (29.7%) in the third trimester. The reported gestational age at diagnosis ranged from 6.7 to 38 weeks. Our patient was diagnosed at six weeks and five days of gestation, which is relatively early among reported cases during pregnancy [5].

Second, systemic embolisation manifested as bilateral renal infarction without evidence of neurological or cardiopulmonary involvement. Emboli originating from cardiac myxomas most commonly affect the cerebral circulation, whereas renal embolisation is uncommon [2,4]. In previously reported cases of cardiac myxoma during pregnancy, symptoms related to intracardiac obstruction were commonly reported, while embolic manifestations were predominantly described in the cerebral, coronary and retinal circulation [5,6,11]. Acute abdominal or flank pain during pregnancy is commonly associated with obstetric, gastrointestinal or urinary tract disorders [8]. In our patient, the combination of severe pain, elevated CRP and LDH levels and unremarkable abdominal ultrasonography prompted further evaluation with non-contrast-enhanced abdominal MRI, which raised suspicion of bilateral renal infarction. The present case highlights the diagnostic challenges associated with atypical embolic manifestations of cardiac myxoma during pregnancy.

Third, the tumour originated from the Coumadin ridge, an uncommon attachment that complicated diagnostic evaluation [3]. In accordance with contemporary multimodality imaging recommendations, transthoracic echocardiography was used as the initial diagnostic modality and demonstrated a mobile left atrial mass. Subsequently, CMR was performed, providing further anatomical and tissue characterisation information and strengthening the suspicion of a left atrial myxoma. Most left atrial myxomas arise from the region of the fossa ovalis of the interatrial septum, whereas alternative sites of attachment are distinctly uncommon [1-3]. Diagnostic uncertainty arose from the atypical attachment to the Coumadin ridge (also known as the Warfarin ridge or left atrial ridge). This structure is a normal anatomical fold between the left superior pulmonary vein and the left atrial appendage, which itself can mimic a tumour or thrombus [3]. FDG-PET/CT was performed to assess for an underlying malignant or lymphoproliferative process. Whether the unusual Coumadin ridge attachment contributed to the embolic presentation in our patient remains uncertain. Evidence on tumour location as a predictor of embolisation is conflicting. An earlier meta-analysis reported atypical tumour location as a significant risk factor for embolic complications, whereas a more recent meta-analysis found no significant association between tumour location and embolic risk. Irregular tumour surface characteristics and high tumour mobility, however, appear more consistently associated with an increased embolic event risk [12,13].

Management of cardiac myxoma during pregnancy requires careful balancing of maternal and foetal risks and should be individualised through multidisciplinary decision-making, ideally within a Pregnancy Heart Team as recommended by the recent guidelines of the European Society of Cardiology for cardiovascular disease and pregnancy [7]. Complete surgical excision is the definitive treatment because therapeutic anticoagulation does not eliminate the risk of tumour-related embolisation [1,2]. Given the limited available evidence, optimal surgical timing remains uncertain [5,6,14]. Reviews and case reports describe different management strategies, ranging from immediate surgery in patients with haemodynamic instability or high embolic risk to postponing surgery until the second trimester in clinically stable patients [5,6,14-16]. At only six weeks and five days of gestation, our patient had already experienced systemic embolisation from the myxoma. Postponing surgery was considered to carry an unacceptably high risk of recurrent embolic events. Following multidisciplinary counselling regarding the foetal risks associated with cardiopulmonary bypass in early pregnancy [7,10] and the maternal risk of recurrent embolisation, the patient opted for termination of pregnancy before definitive cardiac surgery. This case illustrates that management decisions in early pregnancy must integrate gestational age, foetal status, tumour characteristics, embolic risk and the patient's individual preferences.

This report has several limitations. One limitation of this report is that genetic testing for a hereditary myxoma syndrome, including Carney complex, was not performed. The patient had a solitary tumour, and no relevant family history or other clinical features suggestive of Carney complex were documented. Nevertheless, an underlying hereditary predisposition cannot be excluded. The echocardiographic follow-up period was limited to 10 months and therefore does not allow conclusions regarding long-term tumour recurrence. Finally, the original surgical and histopathological reports and the original FDG-PET/CT images were unavailable for independent review; however, the respective findings were documented in the discharge summary.

## Conclusions

Left atrial myxoma may initially present with bilateral renal infarction in the absence of neurological or cardiopulmonary manifestations. Unexplained renal infarctions should therefore prompt evaluation for a cardiac source of embolism, including the possibility of a previously undiagnosed cardiac myxoma. This case highlights the importance of recognising visceral infarction as an atypical manifestation of cardiac myxoma in pregnant patients with unexplained abdominal or flank pain. It further emphasises the importance of multidisciplinary, individualised management of cardiac myxoma during early pregnancy, balancing maternal and foetal risks in terms of tumour characteristics, embolic risk, gestational age and patient preferences.
